# Correlated mRNAs and miRNAs from co-expression and regulatory networks affect porcine muscle and finally meat properties

**DOI:** 10.1186/1471-2164-14-533

**Published:** 2013-08-05

**Authors:** Siriluck Ponsuksili, Yang Du, Frieder Hadlich, Puntita Siengdee, Eduard Murani, Manfred Schwerin, Klaus Wimmers

**Affiliations:** 1 Research Group ‘Functional Genome Analyses, Leibniz Institute for Farm Animal Biology (FBN), Wilhelm-Stahl-Allee 2, D-18196, Dummerstorf, Germany; 2 Institute for Genome Biology, Leibniz Institute for Farm Animal Biology (FBN), Wilhelm-Stahl-Allee 2, D-18196, Dummerstorf, Germany

## Abstract

**Background:**

Physiological processes aiding the conversion of muscle to meat involve many genes associated with muscle structure and metabolic processes. MicroRNAs regulate networks of genes to orchestrate cellular functions, in turn regulating phenotypes.

**Results:**

We applied weighted gene co-expression network analysis to identify co-expression modules that correlated to meat quality phenotypes and were highly enriched for genes involved in glucose metabolism, response to wounding, mitochondrial ribosome, mitochondrion, and extracellular matrix. Negative correlation of miRNA with mRNA and target prediction were used to select transcripts out of the modules of trait-associated mRNAs to further identify those genes that are correlated with post mortem traits.

**Conclusions:**

Porcine muscle co-expression transcript networks that correlated to *post mortem* traits were identified. The integration of miRNA and mRNA expression analyses, as well as network analysis, enabled us to interpret the differentially-regulated genes from a systems perspective. Linking co-expression networks of transcripts and hierarchically organized pairs of miRNAs and mRNAs to meat properties yields new insight into several biological pathways underlying phenotype differences. These pathways may also be diagnostic for many myopathies, which are accompanied by deficient nutrient and oxygen supply of muscle fibers.

## Background

Muscle is the major energy consuming and storage organ. An imbalance of nutrients, energy, and oxygen supply-and-demand in muscle cells is evident following cardiac muscle or skeletal muscle attack, injury, or damage. The consequences of these imbalances depend on muscle structure and metabolism and, thus, the muscle’s entire complement of proteins and their expression patterns. Similar changes, i.e., termination of nutrient and energy supplies and anoxia, also occur in muscle cells *post mortem*. Indeed, these changes underlie the conversion of muscle to meat in food production. The physiological processes accompanying the change of muscle to meat involve expression of many genes associated with muscle structure and metabolic features [1,2]. Genes active in the muscle could therefore potentially have pathogenetic effects by disturbing muscle energy and oxygen homeostasis *in vivo*, as well as conferring traits related to meat quality *post mortem*.

Meat quality is complex and is affected by genetic and environmental factors as well as slaughtering procedures [3]. The conversion of muscle to meat is important not only as an economic factor in pork production, but also because these events mimic pathological processes associated with muscle injury or damage in humans. *Post mortem* traits for meat quality and carcass are influenced by a complex network of gene interactions in muscle; therefore, elucidating the relationships between genes and how these genes, in turn, influence meat quality and carcass traits is critical for developing a comprehensive understanding of the muscle to meat conversion as well as muscle pathologic processes including muscle atrophy, dystrophy, and hypoxia. Additionally, pigs share many genomic and physiological similarities with humans and, therefore, provide a good model to study the genetic determination of complex traits and as a biomedical model [4,5].

Recent advances in functional genomic screening, which can help determine molecular processes underlying phenotypic differences [6-8], have identified roles for microRNAs (miRNAs) in regulation of myogenesis [9-11] and adipogenesis [12-14]. miRNAs are small, non-coding RNA molecules of approximately 22 nucleotides. The primary miRNA transcript has a stem-loop structure that is recognized and cleaved via RNA processing enzymes to produce a double-stranded duplex. miRNAs target mRNA transcripts via base-pair complementarity, typically in the 3′ untranslated region [15,16], but also in the coding sequence [17]. This targeting can induce transcript cleavage, degradation, destabilization, or repression of translation, thereby modulating protein levels. It has been recently shown that reduction of transcript level account for most of the regulatory, repressive effects of miRNAs [18]. Target genes that are regulated by miRNAs through degradation of their respective transcripts consequently show negative correlation of their mRNA with the miRNA regulator. Moreover, one miRNA can target several — even hundreds of — genes. Therefore, a unique approach for identifying miRNA-mRNA regulatory modules was recently introduced, whereby paired miRNA-mRNA expression profiles were constructed to predict putative target genes of miRNAs [19,20].

Many studies used the network analysis for dissecting the complex traits [21,22]. Weighted gene co-expression network analysis (WGCNA) [23] has been successfully applied in a variety of different settings [24-28]. WGCNA groups genes into modules based on their co-expression across a set of samples and finally relates these modules to the traits of interest in order to elucidate relevant modules or genes.

In order to identify groups of co-expressed genes (mRNAs) and on the hierarchically superior level of miRNAs that are correlated with organismal traits related to carcass and meat quality, we first applied weighted gene co-expression network analysis (WGCNA) and subsequently we adapted the paired expression profile approach. We identified co-expression networks regulated by miRNA after filtering of negatively-correlated miRNA-mRNA pairs and predicting target genes. The integration of miRNA and mRNA expression analyses as well as network analysis enabled us to interpret the differentially-regulated genes from a systems perspective, yielding new insight into several biological pathways underlying phenotypic differences.

## Results

### Meat quality and carcass traits phenotypes

Elucidating the relationships between genes and how these genes, in turn, influence muscle metabolic and structural properties is critical for developing a comprehensive understanding of the muscle to meat process as well as muscle pathologic and regenerative processes related to muscle atrophy, dystrophy and hypoxia. In total, 207 performance-tested crossbred pig [PI × (DL × DE)] samples were used to investigate meat quality and carcass traits. Descriptions of 7 carcass traits and 13 meat quality traits, as well as means and standard deviations, analysed in this study are shown in Table 1. High correlation coefficients were found between the same biochemical and biophysical parameters measured at different positions and at different time points *post mortem*, like fatness traits or pH. The other cluster covers the traits drip loss, protein content, and conductivity. This cluster was negatively correlated to pH or fatness (Additional file 1: Figure S1).

**Table 1:** Measured carcass and meat quality traits

**Traits**	**Definitions of traits**	**Mean ± SD (N** = **207)**
loin eye area (LEA) [cm^2^]	area of *M*. *longissimus dorsi* (Mld) at 13th/14th rib	52.96 ± 5.7
fat area (FA) [cm^2^]	fat area on Mld at 13th/14th rib	14.97 ± 3.2
meat to fat ratio (MFR)	ratio of meat and fat area	0.29 ± 0.1
fat depth at shoulder (FDS) [cm]	depth of fat and skin on muscle, mean of 3 measures at thickest point	3.44 ± 0.4
fat depth at tenth rib (FDTR) [cm]	depth of fat and skin on muscle, mean of 3 measures at thinnest point	1.92 ± 0.4
loin fat depth at loin (FDL) [cm]	depth of fat and skin on muscle, mean of 3 measures at thinnest point	1.34 ± 0.4
average back fat (ABF) [cm]	mean value of shoulder fat depth, back fat tenth rib and loin fat depth	2.23 ± 0.4
Drip loss (DL) %	% of weight loss of Mld collected at 24 h *post mortem*, held for 48 h at 4°C	5.37 ± 2.2
LF24MLD	Conductivity in Mld at 13th/14th rib 24 h post mortem	5.35 ± 2.2
LF45MLD	Conductivity in Mld at 13th/14th rib 45 min *post mortem*	4.98 ± 1.6
Intramuscular fat content (MLDIMF) %	Intramuscular fat content of Mld at 13th/14th rib	0.79 ± 0.4
Protein content (MLDP) %	Protein content of Mld at 13th/14th rib	23.65 ± 0.5
Water content (MLDW) %	Water content of Mld at 13th/14th rib	74.7 ± 0.6
Ash content (MLDA) %	Ash content of Mid at 13th/14th rib	1.06 ± 0.1
meat colour (OPTO)	meat colour 24 h *post mortem* in Mld at 13th/14th rib; OPTO star	68.56 ± 6.4
IMP24MLD	Impedance of Mld at 24 h *post mortem*	44.63 ± 15.6
pH45MLD	pH value in Mld at 13th/14th rib 45 min *post mortem*	6.15 ± 0.3
pH24MLD	pH value in Mld at 13th/14th rib 24 h *post mortem*	5.48 ± 0.1
pH45MSM	pH value in *M*. *semimembranosus* (Msm) at 45 min *post mortem*	6.24 ± 0.3
pH24MSM	pH value in *M*. *semimembranosus* (Msm) at 24 h *post mortem*	5.53 ± 0.1

### Gene co-expression network construction for mRNA

To investigate the role of transcriptional networks in muscle, we performed a weighted gene co-expression network analysis (WGCNA) using expression data from *M*. *longissimus dorsi* necropsies of the 207 performance-tested crossbred pigs [PI × (DL × DE)]. Expression analysis using GeneChip Porcine Genome Arrays (Affymetrix) containing 24,123 probe sets identified 11,191 probe sets with consistent expression according to MAS5 analysis; these were used for further analysis. Using WGCNA, residuals derived from the mixed-model analysis of expression levels of 11,191 probe sets were used for constructing the muscle transcriptional network. WGCNA grouped genes into 22 modules based on patterns of co-expression. Each module was labelled with a unique color identifier and was characterized for enrichment of genes of specific gene ontology (GO) categories (Table 2). To represent the gene expression profiles of the highly correlated genes inside a given module, we used the first principal component, which is referred to as the module eigengene (ME). We tested each ME for correlation with meat and carcass traits.

**Table 2:** List of the top GO terms in the most significant DAVID functional clusters for each muscle network module

**Module**	**Top term**	**No. of genes in ME**	**% count**^**1**^	**Top term *P-value***
blue	GO:0044451 ~ nucleoplasm part	524	8.97	1.32E-11
light-green	GO:0044429 ~ mitochondrial part	97	59.79	3.24E-54
dark-orange	GO:0006414 ~ translational elongation	24	41.67	1.37E-14
grey60	GO:0030163 ~ protein catabolic process	105	12.38	3.10E-04
magenta	GO:0046907 ~ intracellular transport	208	10.58	3.43E-05
red	GO:0005761 ~ mitochondrial ribosome	315	5.08	4.41E-16
black	GO:0005739 ~ mitochondrion	436	27.06	4.88E-46
salmon	GO:0006414 ~ translational elongation	137	45.99	4.93E-107
green	GO:0032446 ~ protein modification by small protein conjugation	246	4.07	7.90E-05
dark-grey	GO:0030036 ~ actin cytoskeleton organisation	37	16.22	8.94E-05
tan	GO:0031012 ~ extracellular matrix	154	31.17	7.96E-39
midnightblue	GO:0042060 ~ wound healing	122	7.38	5.38E-05
pink	GO:0000323 ~ lytic vacuole	265	10.57	2.78E-16
dark-turquoise	GO:0006006 ~ glucose metabolic process	31	16.13	3.00E-04
purple	GO:0006986 ~ response to unfolded protein	143	6.38	8.28E-08
light-yellow	GO:0006954 ~ inflammatory response	85	14.12	7.94E-07
orange	GO:0009611 ~ response to wounding	26	23.08	1.37E-03
brown	GO:0031981 ~ nuclear lumen	1436	15.48	2.72E-27
dark-red	GO:0031981 ~ nuclear lumen	183	21.86	8.14E-10
cyan	GO:0044265 ~ cellular macromolecule catabolic process	182	13.19	1.61E-06
dark-green	GO:0019941 ~ modification-dependent protein catabolic process	38	13.16	2.93E-02
grey	GO:0008219 ~ cell death	3616	23.84	7.09E-17

^1^(number of genes in term/number of genes in ME) × 100).

### Module-trait associations of mRNA

**Figure 1:**
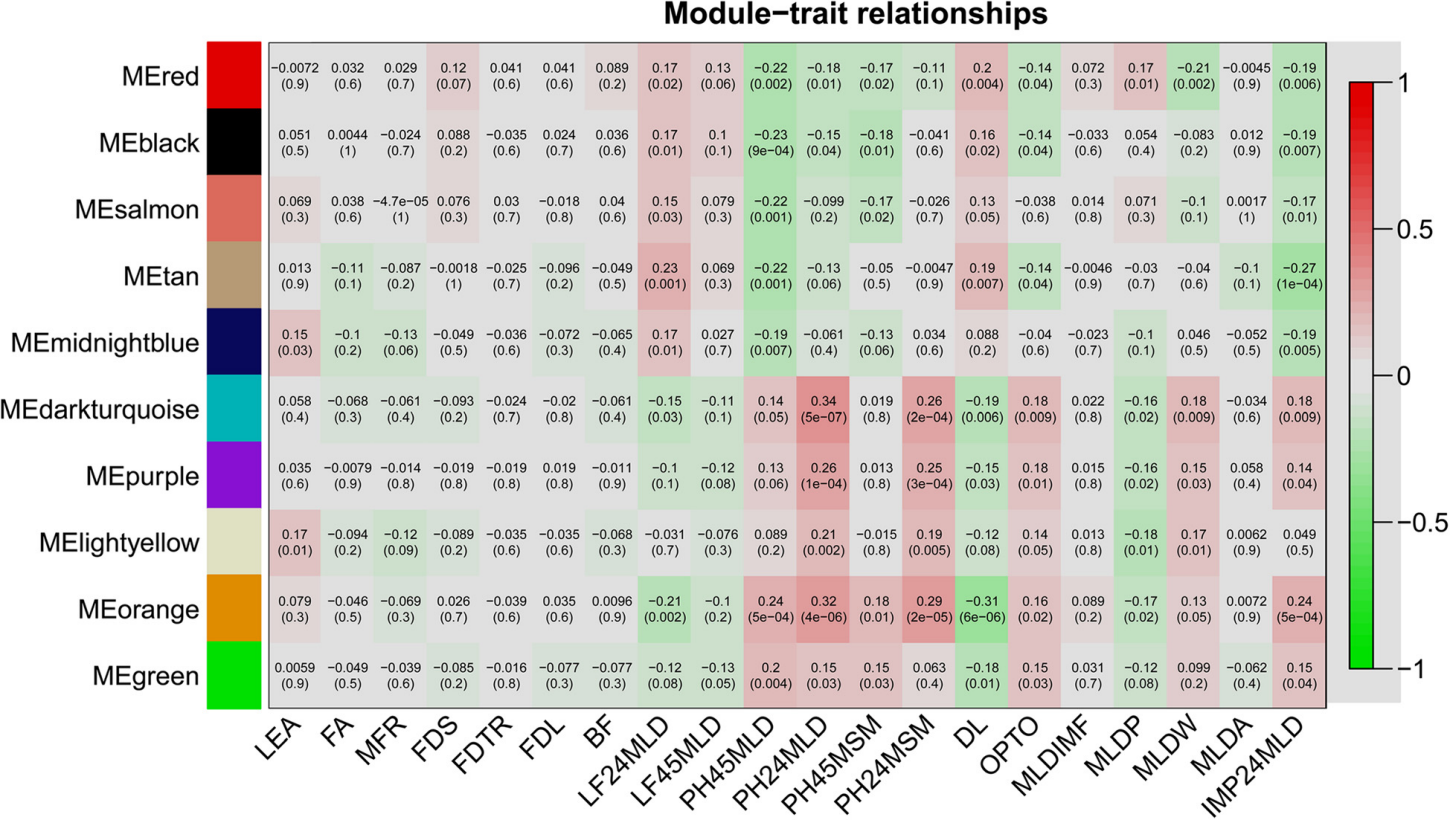
**Correlation matrix of module eigengene values obtained for mRNAs and phenotypes.** Weighted gene co-expression network analysis (WGCNA) groups genes into modules based on patterns of gene co-expression. Each of the modules was labelled with a unique color as an identifier. Twenty-two modules were identified; each module eigengene was tested for correlation with meat and carcass traits. Within each cell, upper values are correlation coefficients between module eigengene and the traits; lower values are the corresponding p-values.

Sets of genes (modules) with common expression patterns that were associated with particular traits were identified based on the correlation between ME and organismal phenotype. We identified five modules that significantly associated with meat quality. Modules dark-turquoise and orange were correlated positively to pH traits and negatively to drip loss (ME_[dark-turquoise]_: pH24MLD r = 0.34, p = 5.3 × 10^-7^, DL r = -0.19, p = 5.6 × 10^-3^; ME_[orange]_: pH24MLD r = 0.32, p = 3.7 × 10^-6^, DL r = -0.31, p = 5.8 ×10^-6^) (Figure 1). Module dark-turquoise (31 annotated genes) was highly enriched for genes belonging to the cluster “glucose metabolic process” (GO: 0006006) and the KEGG-pathway “insulin signaling” with an enrichment score (ES) of 2.65. Module orange (26 annotated genes) was enriched for transcripts of the functional annotation clusters “response to wounding”, “defense response” and “inflammatory response” (ES = 2.42). Modules red, black, and tan were correlated negatively to pH traits and positively to drip loss (ME_[red]_: pH45MLD r = -0.22, p = 1.8 × 10^-3^, DL r = 0.20, p = 3.9 × 10^-3^; ME_[black]_: pH45MLD r = -.23, p = 8.8 × 10^-4^, DL r = 0.16, p = 1.8 × 10^-2^; ME_[tan]_: pH45MLD r = -0.22, p = 1.4 × 10^-3^, DL r = 0.19, p = 6.9 × 10^-3^) (Figure 1). Further, modules red (315 annotated genes), black (436 annotated genes), and tan (154 annotated genes) were enriched for genes of the top functional annotation clusters of “mitochondrial ribosome”, “mitochondrion”, and “extracellular matrix” with ES of 10.23, 15.15, and 27.05, respectively. Only one module (ME_[dark-orange]_) showed association with traits related to fatness (Figure 1).

### Co-expression networks and module-trait associations for miRNA

**Figure 2:**
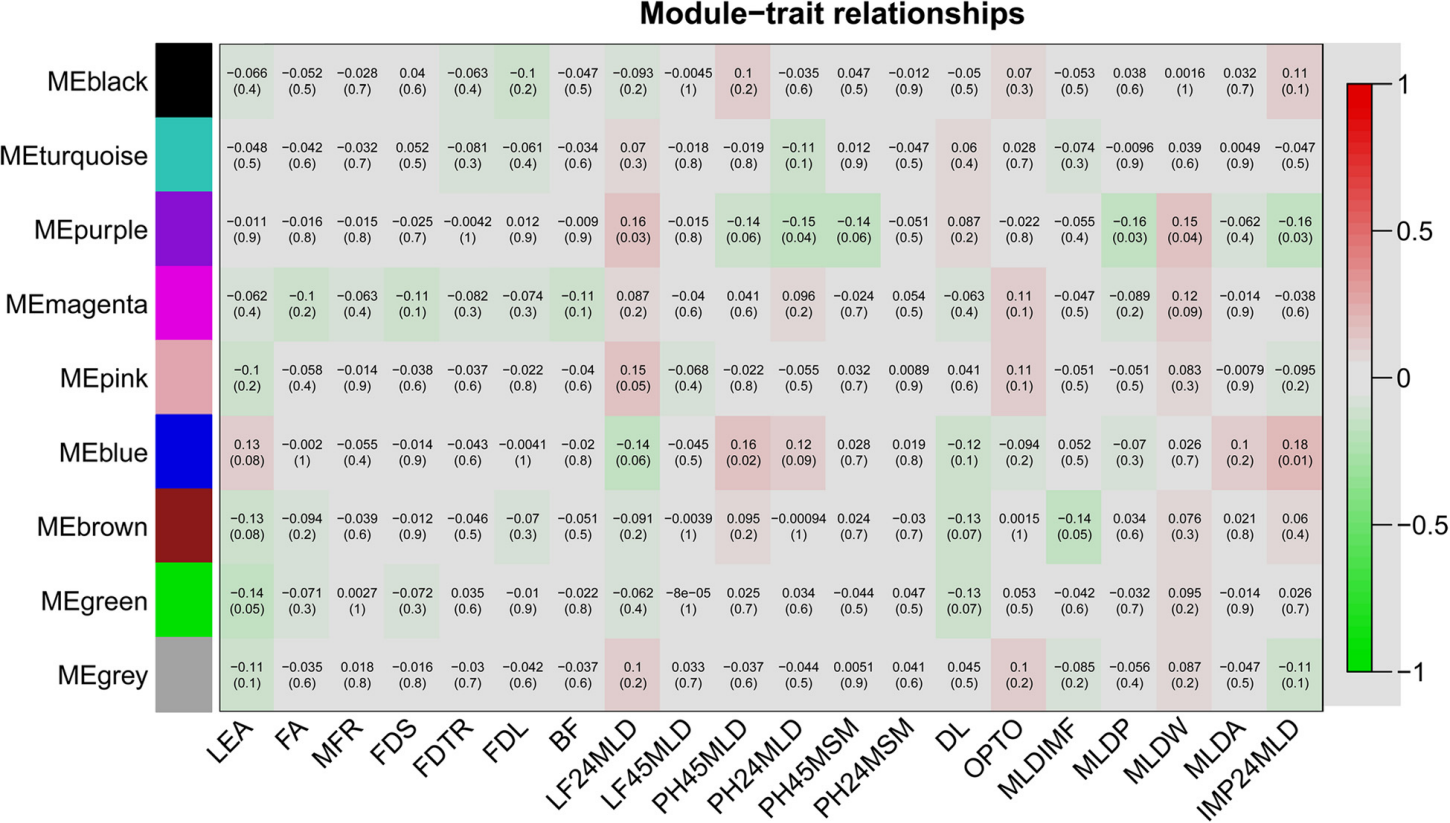
**Correlation matrix of module eigengene values obtained for miRNAs and phenotypes.** Weighted gene co-expression network analysis (WGCNA) groups miRNA into modules based on patterns of their co-expression. Each of the modules was labelled with a unique color as an identifier. Nine modules were identified; each module eigengene was tested for correlation with meat and carcass traits. Within each cell, upper values are correlation coefficients between module eigengene and the traits; lower values are the correspondent p-value.

Transcriptional networks of muscle microRNAs were studied with a WGCNA using miRNA expression data on *M*. *longissimus dorsi* from the same animals as above. Residuals, derived from the analysis of expression levels after correction for systematic effects according to the mixed model, were used for constructing the muscle miRNA transcriptional networks, i.e., modules. Nine modules were identified (Figure 2). Only 2 modules were associated with meat quality at a significance level of p < 0.05. Module purple was correlated positively to LF24MLD at p = 0.03 and negatively to pH24MLD and IMP24MLD at p = 0.04 and p = 0.03, respectively. Module purple consisted of 8 miRNA families (miR-17, miR-30. miR221, miR-185, miR-324, miR362, miR-500, and miR-542). Module blue was positively correlated to pH45MLD and IMP24MLD at p = 0.02 and p = 0.01, respectively. Module blue comprised 29 miRNA families (let-7, miR-15, miR-17, miR-31, miR-95, miR-103, miR-105, miR-122, miR-124, miR-130, miR-138, miR-154, miR-184, miR-185, miR-197, miR-202, miR-204, miR-212, miR-214, miR-320, miR-326, miR-335, miR-346, miR-383, miR-467, miR-491, miR-744, miR-1224, and miR-1296).

### Individual miRNA expression profiles and correlated traits

In addition to miRNA modules, the expression of 675 individual miRNAs probe sets, which corresponded to 513 unique sequences belonging to 159 miRNA families, were profiled and examined for correlation with meat and carcass traits. In total, 225 miRNA-phenotype pairs revealed correlations at p < 0.01. Table 3 lists the top 20 miRNAs with highly significant correlations to phenotypes (p < 0.005).

**Table 3:** Correlation coefficient of carcass and meat quality traits with abundance of individual miRNAs and the positions of miRNAs

**Trait**	**miRNA**_**family**	**r**	**p**-**value**	**Chromosome**	**Position (bp)**
DL	miR_184	-0.23	1.35E-03	7	5.38E + 07
DL	miR_142	0.23	1.77E-03	12	3.59E + 07
DL	miR_23	-0.21	4.42E-03	10	3.29E + 07
DL	miR_181	-0.20	4.62E-03	10	2.74E + 07
IMP24MLD	miR_217	-0.25	6.01E-04	3	8.87E + 07
IMP24MLD	miR_184	0.25	6.41E-04	7	5.38E + 07
IMP24MLD	miR_221	-0.24	8.12E-04	X	4.34E + 07
LF24MLD	miR_221	0.25	4.49E-04	X	4.34E + 07
MLDP	miR_185	-0.21	3.86E-03	14	5.58E + 07
MLDIMF	miR_467	0.24	1.03E-03	9	1.29E + 08
OPTO	miR_1827	0.24	1.03E-03	1	2.33E + 08
PH24MLD	miR_133	-0.27	1.86E-04	6	9.92E + 07
PH24MLD	miR_217	-0.26	2.36E-04	3	8.87E + 07
PH24MLD	miR_181	0.25	4.74E-04	10	2.74E + 07
PH24MLD	miR_130	-0.23	1.26E-03	2	1.31E + 07
PH24MSM	miR_133	-0.24	8.24E-04	6	9.92E + 07
PH24MSM	miR_363	0.23	1.39E-03	X	1.17E + 08
FDS	miR_103	-0.24	6.65E-04	17	3.65E + 07
FDS	miR_107	-0.24	7.63E-04	14	1.11E + 08
FDS	miR_17	-0.24	8.61E-04	X	1.17E + 08

### Endogenous correlation of expression profiles between miRNA and mRNA

We performed pairwise correlation coefficient analysis to evaluate association of expression levels between 675 miRNA probe sets and 11,191 mRNA probe sets. Among the 7,553,925 Pearson correlation coefficients, we detected significant correlation in 5,933 miRNA-mRNA pairs at p-values ≤ 8.97 × 10^-5^ (FDR = 0.1). The 5,933 pairs comprised 408 miRNA probe sets belonging to 128 miRNA families that were correlated with 2,296 mRNA probe sets. Of these 5,933 pairs, 4,005 and 1,928 pairs showed positive and negative correlations, respectively. Positive correlations tended to be more dramatic than negative correlations; the correlation between miR-122 and VTN was the most significant (FDR = 4.33x10^-11^). The most significant negative correlation was between miR-154 and LOC387820 (FDR = 1.1x10^-5^). The most frequently involved miRNA family was miRNA-221, which was correlated with 616 mRNAs. In total 96 miRNA families showed significantly negative correlations with groups of up to 253 genes. We evaluated GO classification for each miRNA-correlated gene set of more than 50 genes (Table 4). The most striking findings were from gene sets that were negatively correlated with miR-23, miR-30, miR17, miR154, and miR-132. For miR-23 and miR-17 the set of negatively correlated genes was highly enriched for genes belonging to the clusters “translation” (GO:0006412) and “translational elongation” (GO:0006414). The set of genes negatively correlated with miR-30 was enriched for “cytoskeletal protein binding” (GO:0008092). The set of genes negatively correlated with miR-154 was enriched for “threonine-type peptidase activity” (GO:0070003), while miR132-correlated genes were enriched for “proteasome complex” (GO:0000502).

**Table 4:** **List of the top GO terms in the most significant DAVID functional clusters of genes negatively**-**correlated with the listed miRNAs**

**miRNA**	**Top term**	**No. of genes**^**1**^	**% count**^**2**^	**Top term *P***-***value***
miR-23	GO:0006412 ~ translation	253	6.14	2.25E-11
miR-30	GO:0008092 ~ cytoskeletal protein binding	96	14.03	1.83E-03
miR-17	GO:0006414 ~ translational elongation	87	24.69	1.60E-25
miR-154	GO:0070003 ~ threonine-type peptidase activity	72	5.88	3.98E-05
miR-132	GO:0000502 ~ proteasome complex	64	4.84	1.63E-02

^1^number of genes with negative correlation to the respective miRNAs.

^2^(number of genes in term/number of genes in ME) × 100).

### Integration of negative correlation of miRNA and mRNA with module-trait association and target prediction

**Figure 3:**
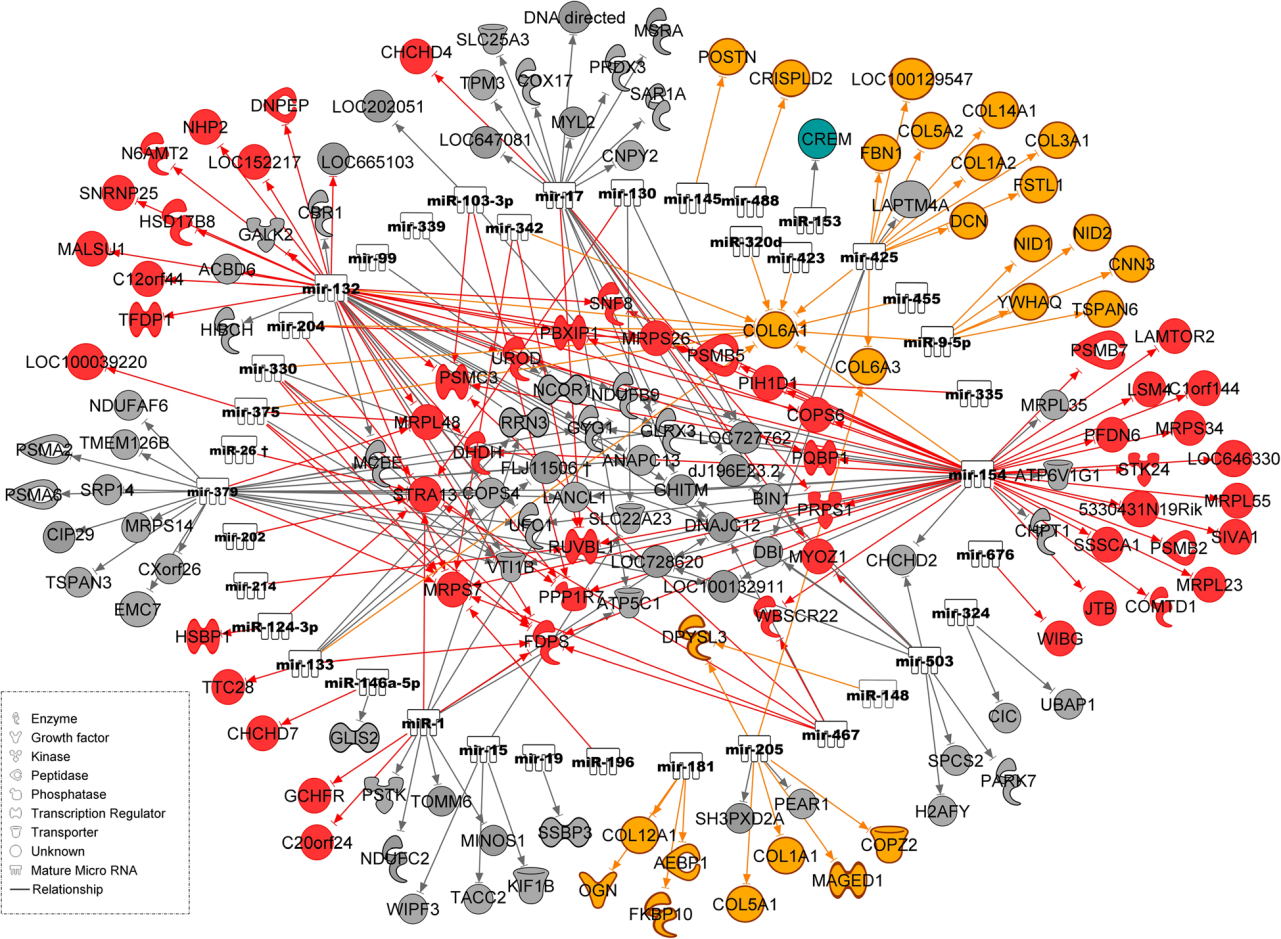
**Regulatory network of negatively**-**correlated mRNAs and miRNAs.** Genes in modules dark-turquoise, red, black, and tan that were significantly associated with meat quality and were negatively correlated with various miRNAs as indicated by the arrows. Colors of symbols of mRNA encoded proteins indicate the assignment to the respective module (grey = black).

**Figure 4:**
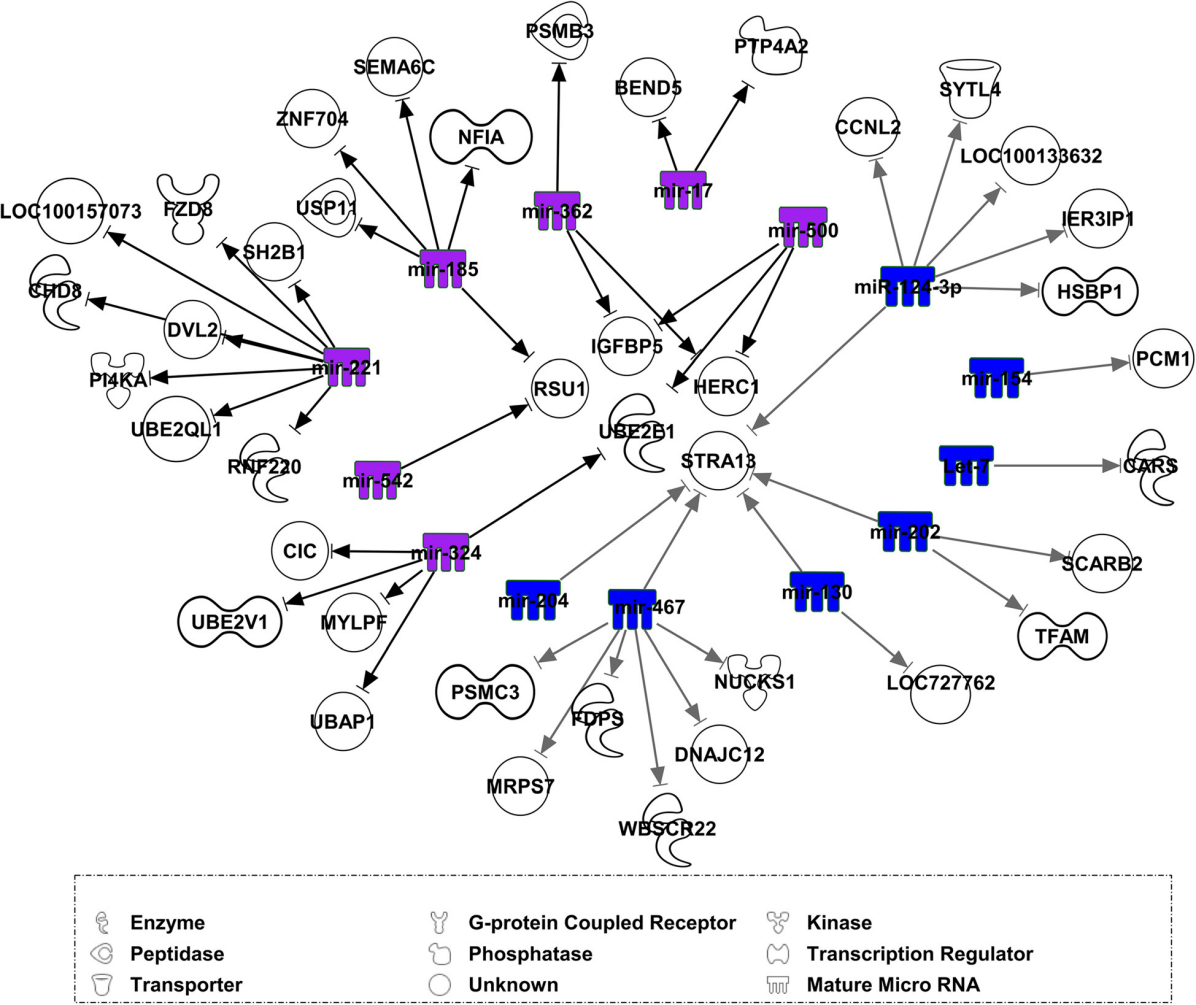
**Regulatory network of negatively**-**correlated miRNAs and mRNAs.** miRNAs in modules blue and purple that were significantly associated with meat quality and were negatively correlated with various mRNAs as indicated by the arrows. Colors of symbols of miRNAs indicate the assignment to the respective module.

A total of 1,928 pairs of miRNAs and mRNAs that showed negative correlations at p-values ≤ 8.97 × 10^-5^ (FDR = 0.1) belonged to 1,073 mRNA probe sets (929 gene) and 264 miRNA probe sets (96 miRNA families). Of these, 286 pairs were assigned to modules dark-turquoise, red, black, and tan, which showed correlation with traits related to meat quality (Figure 3). However, no genes in module orange were negatively correlated with miRNA at FDR < 0.1. Only one gene (*CREM*) in the module dark-turquoise was negatively correlated with miR-153 (r = -0.34 p = 2.11x10^-06^ FDR = 0.02). In module black, 69 genes were negatively correlated with 21 miRNA families, totaling 140 miRNA-mRNA pairs. In total, 101 and 43 pairs of miRNAs and mRNAs were identified in modules red (22 miRNAs and 52 genes) and tan (17 miRNAs and 26 genes). Known genes belonging to modules dark-turquoise, red, black, and tan and their negatively-correlated miRNAs are shown in Table 5. Out of 1,928 pairs of miRNAs and mRNAs that showed negative correlations at p-values ≤ 8.97 × 10^-5^ (FDR = 0.1), 62 pairs were assigned to modules blue and purple, which correlated with meat quality (Figure 4). These 62 pairs of miRNAs and mRNAs belonged to 14 miRNA families and 40 genes (Table 6).

**Table 5:** **Genes belonging to the modules dark**-**turquoise**, **red**, **black**, **and tan and their negatively**-**correlated miRNAs**

**Modules**	**miRNA family**	**Genes within modules negatively correlated with miRNA**^**1**^
Dark-turquoise	miR-153	*CREM*
Black	miR-1	*NDUFB9*, *ATP5C1*, *DNAJC12*, *NDUFC2*, *TOMM6*, ***RRN3***, *PSTK*, *C1orf151*
	miR-103	*GLRX3*, *LOC202051*
	miR-130	***SLC22A23***, *LOC727762*
	miR-132	*NDUFB9*, *ACBD6*, *GHITM*, *DNAJC12*, *GLRX3*, ***CBR1***, *ANAPC13*, *RRN3*, *HIBCH*, *MCEE*, *GYG1*, *UFC1*, *COPS4*, *GALK2*, *LOC665103*, ***VTI1B***
	miR-133	***RRN3***, *COPS4*, *FLJ11506*
	miR-146	*GLIS2*
	miR-15	***KIF1B***, *TACC2*, *Wipf3*, *SLC22A23*
	miR-154	*NDUFB9*, ***GHITM***, *GLRX3*, *LOC100132911*, *CHPT1*, *GYG1*, *UFC1*, *LANCL1*, *LOC728620*, *ATP6V1G1*, *MRPL35*, *VTI1B*, *CHCHD2*
	miR-17	*SLC25A3*, *PRDX3*, *GHITM*, ***SAR1A***, *ATP5C1*, *COX17*, *BIN1*, *GLRX3*, *MYL2*, *TPM3*, *LOC647081*, *CNPY2*, ***dJ196E23***.***2***, ***LOC727762***, *MSRA*
	miR-181	
	miR-19	*SSBP3*
	miR-205	*SH3PXD2A*, *PEAR1*
	miR-324	***CIC***, ***UBAP1***
	miR-330	*DNAJC12*, ***VTI1B***
	miR-339	***Ncor1***
	miR-375	***VTI1B***
	miR-379	*C15orf24*, *GHITM*, *CIP29*, *TSPAN3*, ***GLRX3***, ***PSMA2***, *ANAPC13*, *C8orf38*, *SRP14*, *CXorf26*, *LOC100132911*, *MRPS14*, *MCEE*, *GYG1*, *UFC1*, ***LANCL1***, ***LOC728620***, *COPS4*, ***PSMA6***, *FLJ11506*, *VTI1B*, *TMEM126B*
	miR-425	*BIN1*, *DBI*, *LAPTM4A*
	miR-467	*DNAJC12*
	miR-503	*SPCS2*, *GHITM*, ***BIN1***, *PARK7*, *DBI*, *LOC728620*, ***H2AFY***, ***dJ196E23***.***2***, ***CHCHD2***
	miR-99	*Ncor1*
Red	miR-1	*FDPS*, *GCHFR*, *C20orf24*, *STRA13*
	miR-103	*PPP1R7*, ***PSMC3***
	miR-124	***HSBP1***, ***STRA13***
	miR-130	***STRA13***
	miR-132	***SNRNP25***, ***PPP1R7***, *PSMC3*, ***FDPS***, *N6AMT2*, *MRPS26*, *PSMB5*, *Snf8*, ***DHDH***, *COPS6*, *HSD17B8*, *PQBP1*, *MRPS7*, *C7orf30*, *TFDP1*, *NHP2*, ***LOC152217***, *PBXIP1*, *DNPEP*, ***C12orf44***, ***UROD***, ***MRPL48***
	miR-133	*FDPS*, ***TTC28***
	miR-146	*CHCHD7*
	miR-154	***PSMB2***, *WBSCR22*, *PPP1R7*, ***PSMC3***, *LSM4*, *FDPS*, *PIH1D1*, *MRPS26*. *5330431N19Rik*, *PSMB5*, *Snf8*, ***JTB***, *DHDH*, *COPS6*, ***PSMB7***, *LOC646330*, *MRPS34*, ***PQBP1***, ***SIVA1***, ***MRPS7***, *STK24*, *RUVBL1*, *MRPL23*, *PFDN6*, *SSSCA1*, *PBXIP1*, *PRPS1*, *MRPL55*, *COMTD1*, *UROD*, *ROBLD3*, *C1orf144*
	miR-17	*MYOZ1*, *CHCHD4*, *RUVBL1*, *PRPS1*
	miR-196	*MRPS7*
	miR-202	*STRA13*
	miR-204	*MRPS26*, ***STRA13***
	miR-214	***RUVBL1***
	miR-26	*STRA13*
	miR-330	***PPP1R7***, ***FDPS***, ***MRPS7***
	miR-335	*PIH1D1*
	miR-342	***MRPS7***, *RUVBL1*
	miR-375	***FDPS***, ***LOC100039220***, ***MRPS7***, *MRPL48*
	miR-379	*MRPS7*, *MRPL48*
	miR-467	*WBSCR22*, *PSMC3*, *FDPS*, *MRPS7*, *STRA13*, *STRA13*
	miR-503	*MYOZ1*
	miR-676	*WIBG*
tan	miR-132	***COL6A1***
	miR-133	*COL6A1*
	miR-145	***POSTN***
	miR-148	***DPYSL3***
	miR-154	*COL6A1*
	miR-181	*COL12A1*, *FKBP10*, ***Aebp1***, *OGN*
	miR-204	*COL6A1*
	miR-205	***COL1A1***, *COL6A3*, ***DPYSL3***, ***MAGED1***, *DPYSL3*, ***COPZ2***, *COL5A1*
	miR-320	***COL6A1***
	miR-330	***COL6A1***
	miR-342	*COL6A1*
	miR-375	***COL6A1***
	miR-423	***COL6A1***
	miR-425	*DCN*, *COL3A1*, ***FBN1***, *COL6A3*, *LOC100129547*, *COL5A2*, *COL1A2*, ***COL6A1***, *FSTL1*, ***COL14A1***
	miR-455	*COL6A1*
	miR-488	*CRISPLD2*
	miR-9	***NID2***, *COL6A1*, *YWHAQ*, *CNN3*, *NID1*, *TSPAN6*

^1^genes predicted as potential targets of the corresponding miRNAs are shown in bold.

**Table 6:** **MicroRNAs belonging to modules blue and purple and their negatively**-**correlated genes**

**Modules**	**miRNA family**	**Genes within modules negatively correlated with miRNA**^**1**^
Blue	Let-7	***CARS***
	miR-124	***STRA13***, *IER3IP1*, ***HSBP1***, ***CCNL2***, *LOC100133632*, *SYTL4*
	miR-130	***STRA13***, *LOC727762*
	miR-154	*PCM1*
	miR-202	*SCARB2*, *STRA13*, *TFAM*
	miR-204	***STRA13***
	miR-467	*STRA13*, *MRPS7*, *STRA13*, *NUCKS1*, *PSMC3*, *FDPS*, *DNAJC12*, *WBSCR22*
Purple	miR-17	*BEND5*, *PTP4A2*, *BEND5*
	miR-185	*SEMA6C*, *Nfia*, *RSU1*, *USP11*, *SEMA6C*, ***ZNF704***
	miR-221	*RNF220*, *CHD8*, *PI4KA*, ***FLJ25076***, *DVL2*, ***FZD8***, ***Sh2b1***, *LOC100157073*
	miR-324	***CIC***, ***UBE2E1***, ***UBE2V1***, ***MYLPF***, ***UBAP1***
	miR-362	***IGFBP5***, *HERC1*, *PSMB3*
	miR-500	***IGFBP5***, *UBE2E1*, *HERC1*
	miR-542	*RSU1*

^1^genes predicted as potential targets of the corresponding miRNAs are shown in bold.

Further, TargetScan and RNAhybrid were used to scan miRNA and mRNA sequences (porcine RefSeq), to obtain additional evidence for their functional link; these sequences corresponded to 1,928 pairs of negatively-correlated miRNAs and mRNAs. In total, 474 pairs of miRNA and mRNA were confirmed by either of the two *in silico* prediction methods: 331, 195, and 32 miRNA-mRNA pairs were predicted by RNAhybrid, TargetScan, or both methods, respectively. The 474 miRNA-mRNA pairs covered 121 probe sets of miRNAs (65 miRNA families) and 331 targets probe sets (297 genes). When focusing on genes of the trait-correlated modules dark-turquoise, orange, red, black, and tan, 73 out 286 pairs of miRNAs and mRNAs were confirmed with at least one *in silico* method. These 73 pairs comprised 26 miRNA families and 51 genes (Tables 5 and 6 in bold).

## Discussion

Here, we present an integrative approach to identify transcriptomic differences that may contribute to variation of the kinetics of metabolic processes under diminished oxygen and nutrition supply that is evident during muscle conversion to meat. The speed and extend of the switch from aerobic to anaerobic ATP production, until final total failure of energy production, and of protein degradation processes largely affect meat quality [29]. In order to identify functional networks of genes contributing to these processes an approach was used based on a multi-level integration of weighted gene co-expression network analysis (WGCNA) of mRNA and miRNA with mRNA-miRNA pair correlation and miRNA target prediction.

### mRNA abundance and co-expression networks linked to muscle and meat properties

We used transcriptional network analysis to identify co-expression modules (dark-turquoise, orange, red, black, and tan) that correlated to meat quality phenotype. These modules were highly enriched for genes involved in “extracellular matrix”, “glucose metabolic process”, and “mitochondrion” (“oxidative phosphorylation” KEGG_PATHWAY); i.e. processes affecting structural and metabolic properties.

A dominant role of mitochondria is the production of ATP by oxidative phosphorylation that depends on oxygen supply. When oxygen is limited (*post mortem* or during prolonged vigorous exercise) the glucose metabolism occurs by anaerobic respiration, a process that is independent of the mitochondria. A shift from aerobic to anaerobic metabolism - favouring the production of lactic acid - results in a pH decline *post mortem* and thereby influence the meat quality [29]. So the biological process of mitochondria as well as the way of glucose metabolism play a significant role in the muscle cell and finally impact on meat quality. Indeed, mitochondrial dysfunction resulting in decreased cellular energy production is also responsible for a variety of human myopathies and cardiomyopathies [30-33].

Genes assigned to the GO category of “extracellular matrix” encode proteins belonging to the myofibrillar scaffold. The characteristics of the myofibrillar scaffold and the kinetics of their fragmentation were associated with tenderness and water-holding capacity of meat. In particular, the proteolysis of muscle proteins affects the shrinkage of myofibrils, the development of pores in the cell membranes, so called drip channels, and the non-covalent binding of water molecules [34,35]. Collagens are major constituents of the extracellular matrix (ECM). In our study there were many collagen genes that are reported to be correlated with various muscle disorders [36-39]. For example, collagen type VI (COLVI), an important component of skeletal muscle ECM, is involved in maintaining tissue integrity [40]. *Col6a1*^-/-^ mice show a complete absence of collagen VI chains and display a myopathic phenotype, abnormal mitochondria, and increased apoptosis of muscle fibres [41,42].

### miRNA abundance and co-expression networks linked to muscle and meat properties

Our finding of the relevance of mitochondrial metabolic pathways, including oxidative phosphorylation, and muscle structural protein composition to *post mortem* processes affecting meat quality is in line with our previous results obtained in other populations [1,6,7]. Additionally, while the previous studies focused on trait-associated mRNA expression, here another hierarchical level in the regulatory network relevant to processes occurring under conditions of insufficient oxygen, energy, and nutrient supplies is provided. In fact, miRNA was integrated into this study as a regulator molecule of muscle transcripts. miRNAs with identical seed sequences (the same family) [43] or that are closely located on the same chromosome (the same cluster) [44,45] have similar expression trends. This was confirmed in our study, where most of the modules consisted of the same families of miRNAs or miRNAs located on the same chromosome. In this study, marginal association of miRNA co-expression modules to organismal traits was found compared to mRNA co-expression modules. On the one hand, co-expressed miRNAs of the same family or cluster might not regulate the same trait. On the other hand, this may be caused by indirect regulation of organismal traits by miRNAs via their effect on mRNA transcripts. Accordingly, individual miRNA correlation to phenotypes was also considered. Recent studies have revealed key roles for miRNAs in the regulation of skeletal muscle differentiation, and changes in miRNA expression are associated with various skeletal muscle disorders [46-48]. In this study, several miRNAs were correlated with carcass and meat quality traits. This includes miR-221, previously identified in studies of myotube maturation and in the maintenance of the myofibrillar organization [49] and found to contribute to muscle pathogenetic mechanisms [50]. Interestingly, miR-133, which showed highest correlation with pH24MLD and pH24MSM, has been widely studied for roles in the regulation of skeletal muscle development, including in proliferation and myogenesis [10,51] as well as muscle disorders [47]. Recently, a study reported that mice with genetic deletions of miR-133a-1 and miR-133a-2 developed adult-onset centronuclear myopathy in type II (fast-twitch) myofibres, which was accompanied by impaired mitochondrial function, fast-to-slow myofibre conversion, and disarray of muscle triads [52]. These are changes of muscle structure and metabolism that also impact meat quality. In addition to its well established role in translation (Table 4), miR-23a was also recently identified as a key regulator of skeletal muscle differentiation and is predicted to target multiple adult fast myosin heavy chain (Myh) genes, including *Myh1*, *2*, and *4*[53]. For fat traits, miR-103 and miR-107 were highly correlated (Table 3). This is consistent with previous reports of miR-103 being involved in adipogenesis, lipid metabolism, and adipocyte differentiation [54,55] and of miR-103/107 being involved in glucose homeostasis and insulin sensitivity [56].

In this study we showed for the first time more complex correlations among miRNAs and between miRNAs and *post mortem* organismal phenotypes in swine, while also confirming previous studies in human and mouse muscle as well as C2C12 myoblasts.

### Links between miRNA and mRNA that relate to muscle and meat properties

This study also sought to evaluate to what extent the co-expression modules of trait-associated mRNAs are themselves regulated by miRNAs. A regulatory link between miRNA and mRNA and a functional link to the organismal phenotype was suspected if (1) the mRNA belonged to either one of the co-expression modules associated with the traits (i.e., ME_dark-turquoise_, ME_orange_, ME_red_, and ME_tan_), (2) mRNA abundance was significantly negatively correlated with its miRNA regulator, and (3) the mRNA was predicted to be a target gene of the respective miRNA. Therefore, RefSeqs of the genes with present calls from the 3′-IVT-Affymetrix arrays were explored to predict the targets of miRNAs by either seed sequence complementarity [57] or by thermodynamics-based modeling of RNA:RNA duplex interactions [58]. Currently, no publicly-accessible database covers porcine miRNAs and their predicted target genes. Moreover, annotation of porcine genes is not yet finalized. Accordingly, the target predictions should be interpreted with caution.

Interestingly, no miRNA regulator was identified by negative correlation or target prediction in the module orange, which was enriched for genes related to “response to wounding”, “inflammatory response”, and “defense response”. Genes assigned to biofunctions related to response to exogenes stimuli, change their transcription rate immediately due to many factors. As suggested by finding no correlated miRNAs – their regulation of transcription may occur without major involvement of miRNA. However, many genes in the module orange were previously confirmed as transcriptional regulators in myogenesis or were located in QTL regions for muscle fiber traits like BTG2, EGR1, ANKRDS1 and FOS [59,60]. Interestingly, the genes in module orange like Egr1, FOS and JUN that are associated with oxidative stress response were found upregulated in muscle in response to mechanical ventilation and immobilization in a porcine model for critical illness myopathy (CIM) [61,62].

Co-expressed genes in module dark-turquoise were significantly associated with meat quality and based on the current knowledge of gene functions some links among them are suggested to be relevant For example, one member of this module, *CREM*, is a transcription factor binding to cAMP-responsive elements (CREs) in the promoters of various genes. This transcription factor plays important roles in various organismal functions [63-67]. Crem inactivation or knockout has been shown to increase the rate of apoptosis in testis tissue [68,69]. The main cellular change associated with apoptosis processes also occur during post-mortem [70]. Post mortem biochemical processes in muscle lead to pH decline. A high expression of CREM being positively correlated with pH at 24 hours, may indicate a slowdown of apoptosis related post mortem processes paralleling anaerobic metabolic processes that led to a decrease of pH. This indicates that the abundance of CREM transcripts in muscle plays a significant role in meat quality. Further, CREM and miR-153 were highly negatively correlated (FDR < 0.01) which is known to induce apoptosis in a glioblastoma cell line DBTRG-05MG [71]. Thus a consistent link of effects on apoptosis and mRNA-miRNA interaction is obvious. miR-153 also inhibits the protein kinase B (PKB/Akt) pathway by reducing the protein level of insulin receptor substrate-2 (IRS2) [72]. As recently shown, miR-135a targets IRS2 levels by binding to its 3′UTR and this interaction regulates skeletal muscle insulin signaling [73]. Insulin signaling plays a pivotal role in the regulation of glucose uptake by skeletal muscle [74]. The glucose uptake in skeletal muscle has large effects on meat characteristics [75,76]. In our study, Irs-2 also belonged to module dark-turquoise and was negatively correlated with waterholding capacity related traits like DL (p = 0.004) and positively with pH (pH24MLD, p = 7.05E-07; pH24MSM, p = 1.30E-03; pH45MLD, p = 3.39E-03). Thus another plausible functional link of members of the module dark-turquoise and miRNAs can be shown.

Mitochondria supply energy for physiological functions and play a significant role in the regulation of other cellular events including apoptosis, calcium homeostasis, and production of reactive oxygen species. Mitochondrial metabolism is affected by miRNA regulation [77]. Here, we found many miRNAs being negatively correlated to target genes of modules red, black, which were enriched for genes related to mitochondrial pathways. Indeed, miR-338 modulates energy metabolism, oxidative phosphorylation, and mitochondrial functions [78,79], and miR-15b, -16, -195, and -424 decrease cellular ATP levels in cardiomyocytes [80]. Additionally, miR-181c can enter and target the mitochondrial genome, ultimately causing electron transport chain complex IV remodeling and mitochondrial dysfunction [81]. Here we found miR-181 was correlated with DL and pH24MLD as well as S100 calcium binding protein A6 (S100A6). Further, mitochondrial genes like *Kif1b*, *Atp6v1g1*, *Atp5c1*, *Park7*, *Chchd2*, *Ruvbl1*, *Mrps7*, and *Mrpl48* were highly negatively correlated with, and some of them were predicted as targets of, miR-15, -154, -17, -503, -214, -330, -342, and -375.

Many genes of modul tan were assigned in the GO category “extracellular matrix” including *Col1a1*, *Col1a2*, *Col3a1*, *Col5a1*, *Col5a2*, *Col6a1*, *Col6a3*, *Col12a1*, *Col14a1*, *Crispld2*, *Ddn*, *Fbn1*, *Nid1*, *Nid2*, *Ogn*, and *Postn*. *These genes* were negatively correlated with, and some of them were predicted as targets of, miRNA. Interestingly, *Col6a1* was found as a target for many miRNAs including miR-132, miR-205, miR-320, miR-330, miR-375, miR-423, and miR-425. Moreover, miR-205 was identified as a regulator of *Col1a1*, *Maged1*, and *Dpysl3*. Gandellini et al. (2012) [82] reported that miR-205 controls the deposition of laminin-332 and its receptor integrin-β4 as well as participates in a network involving ΔNp63α, which is essential for maintenance of the basement membrane in prostate epithelium. Similarly, other miRNAs, including miR-29, miR-133, and miR-30, are involved in the regulation of development and maintenance of extracellular matrix of bone and muscle [83,84].

Much evidence suggests that a group of miRNAs (cluster and family) may contribute to the regulation of a set of common targets [46,85,86], and are, therefore, associated with phenotypes. WGCNA was used here to group miRNA products and revealed 2 modules associated with meat quality (purple and blue). The miRNA from these modules were negatively correlated to mRNAs, and some of these were predicted as targets. Most miRNAs in module purple were related to genes in the categories “ubiquitin” or “protein catabolic process” (*Ube2e1*, *Ube2b1*, *Ubap1*, *Igfbp5*, *Herc1*, *Psmb3*, *Flj25076*). Differential expression of genes of the ubiquitin system depending on muscle and meat quality was previously shown; only recently also association of genes of the ubiquitin system with meat quality was reported [1,87]. In particular, miR-324 was highly negatively correlated and predicted to target *Ube2e1*, *Ube2v1*, *Bap1*, *Lpf* and *Cic*.

Most previous muscle mRNA and miRNA expression studies focused on cardiac muscle or skeletal muscle injury [50,88]. In these injuries, the degree of damage results from an imbalance of energy, nutrients, and oxygen supply-and-demand in muscle cells. Similarly, nutrient, energy, and oxygen depletion occurs *post mortem*. Many changes in expression associated with muscle injury would therefore overlap with *post mortem* processes in conversion of muscle to meat, and vice versa. In this regard, functional annotation of mRNA co-expression and trait-correlated expression identified key *post mortem* pathways and functions, including glucose metabolic process, mitochondrial metabolic pathways, and muscle structural components, involved in muscle-to-meat conversion that will be relevant to muscle injury as well.

## Conclusion

In this study, for the first time, expression and co-expression of miRNAs—functioning as a fine-tuning of mRNA transcription and translation—was integrated with mRNA transcript abundance measures and phenotypic data on meat quality. By this an additional hierarchical level, i.e. miRNA affecting mRNA, was considered in the molecular regulation of muscle-to-meat conversion. miRNAs are necessary for proper skeletal and cardiac muscle development and function, and have a profound influence on multiple myopathies, such as hypertrophy, dystrophy, and conduction defects. Consequently, an expression biomarker panel (whether from mRNA or miRNA) derived from this study may not only be predictive for quality of meat *post mortem*, but also for many muscle pathologic processes including muscle atrophy, dystrophy, and hypoxia [89,90]. The abundance of mRNAs and their fine-tuning by corresponding miRNAs in molecular pathways related to mitochondrial metabolic balance and oxidative stress, cell proliferation and differentiation, as well as muscle structural protein composition play an important role in these myopathies and meat maturation. Functional studies of the interactions among and between mRNAs and μRNAs will provide additional experimental data for validation of the relationships on the level of mRNAs, miRNAs and organismal phenotype that were stressed in this study.

## Methods

### Animals, tissue collection, and phenotyping

Animal care and tissue collection procedures followed the guidelines of the German Law of Animal Protection, and the experimental protocol was approved by the Animal Care Committee of the FBN. This study was based on trait measurement and expression profile association analyses done with 207 performance-tested pigs from commercial herds of the crossbreed Pietrain × (German Large White × German Landrace). Animals were raised and slaughtered under standardized conditions in the experimental facilities of the Leibniz Institute for Farm Animal Biology (FBN). Sample collection was performed thoroughly after exsanguination, tissue samples were rapidly dissected, snap-frozen in liquid nitrogen and stored at -80°C. The average age of the pigs at sampling was ~180 days. Technological parameters of meat quality, i.e., pH-value, conductivity, and colour, were measured by using Star-series equipment (Rudolf Matthaeus Company, Germany). Measures of pH and conductivity were at 45 min *post mortem* (pH45) and 24 h *post mortem* (pH24), in both *M*. *longissimus dorsi* between 13th/14th rib (pH45MLD, pH24MLD, LF45MLD, LF24MLD) and the ham (*M*. *semimembranosus*) (symbol: pH24MSM, LF24MSM). Muscle colour was measured at 24 h *post mortem* by Opto-Star (Matthaeus, Klausa, Germany). Drip loss was scored based on a bag method with a size-standardized sample from the *M*. *longissimus dorsi* collected at 24 h *post mortem* and weighed, suspended in a plastic bag, held at 4°C for 48 h, and re-weighed [91]. To determine cooking loss, a loin cube was taken from the *M*. *longissimus dorsi*, weighed, placed in a polyethylene bag, and incubated in water at 75°C for 50 minutes. The bag was then immersed in flowing water at room temperature for 30 minutes, and the solid portion was re-weighed. Thawing loss was determined similarly after at least 24 h freezing at -20°C. Drip loss, cooking loss, and thawing loss were calculated as a percentage of weight loss based on the start weight of a sample. Shear force was measured using Instron-4310 equipment, and average values of four replicates were used for analyses.

### Customized miRNA microarrays design

Our custom porcine miRNA array was designed from 284 pig miRNAs obtained from the miRBASE (miRBase 14.0). Because miRNAs are highly conserved between closely-related species [10], we could predict novel porcine miRNA candidates by inter-species alignments requiring 100% mature miRNA similarity. Accordingly, we used previously known miRNA sequences from humans and mice, as well as other species, to perform BLAST searches against the porcine genome database porcine; 391 miRNA candidates were identified. In total, 675 miRNAs probe sets, corresponding to 513 unique sequences belonging to 159 miRNA families, were used for hybridisation with the target samples described above. Microarray data related to all samples were deposited in the Gene Expression Omnibus public repository (GEO accession number: GSE41294: GSM1013731-GSM1013920).

### Customized microarrays, pre-processing, and normalization of miRNA

Total miRNA was isolated with Qiagen miReasy Mini kit and RNeasy MinElute Clean up kit (Qiagen, Hilden, Germany) according to manufacturer’s protocol for small RNA. Quality and quantity of isolated total RNA and miRNA were determined using an Agilent 2100 Bioanalyzer for RNA (Agilent Technologies, Santa Clara, CA). Affymetrix customized microarrays from our porcine miRNA candidate dataset were used. Targets for hybridisation were prepared from miRNA with the FlashTag™ Biotin RNA Labeling Kit for Affymetrix GeneChip miRNA arrays (Genisphere, Hatfield, PA, USA) according to manufacturer’s recommendations. Briefly, 250 ng of miRNA of each individual were poly(A)-tailed using ATP–poly-A-Polymerase, then FlashTag Biotin end-labelled. After hybridisation of biotin-labelled complementary RNA, chips were washed and processed to detect biotin-containing transcripts by Streptavidin-PE (Phycoerythrin) conjugate, then were scanned on GeneChip scanner 3000 7G (Affymetrix, Santa Clara, US). Data were extracted from the images, and spots were quantified and processed by quality filtering. Hybridisation quality was assessed in all samples by using JMP Genomics 5 utilising Robust Multi-array Average (RMA) background correction and log2 transformations. To acquire the expression value, data were normalized between chips using the quantile normalization method.

### Whole-genome expression profiling (mRNA)

Gene expression profiling of *M*. *longissimus dorsi* samples of pigs was conducted with the same animals (207) as for miRNA. In brief, total RNA was isolated using TRI Reagent (Sigma, Taufkirchen, Germany) and used for target preparation for microarray hybridisation. According to Affymetrix protocols, 500 ng of total RNA were reverse-transcribed into cDNA, transcribed into cRNA, and labelled using Affymetrix One cycle synthesis and labelling kit (Affymetrix, UK) to prepare antisense biotinylated RNA targets. Quality of hybridisation was assessed in all samples following manufacturer’s recommendations. Data were analysed with the Affymetrix GCOS 1.1.1 software, using global scaling to a target signal of 500. Data were processed with MAS5.0 to generate cell intensity files (present or absent). Quantitative expression levels of present transcripts were estimated using the PLIER algorithm (Probe Logarithmic Intensity Error) for normalization that was implemented in Expression Console (Affymetrix). Based on BLAST comparison of the Affymetrix porcine target sequences with the porcine genome sequence (Ensembl_Sscrofa_10), 20,689 of the 24,123 probe sets on the Affymetrix Porcine GeneChip were localized and annotated [92,93]. Microarray data related to all samples were deposited in the Gene Expression Omnibus public repository [GEO accession number: GSE32112: GSM796045-GSM796251].

### Pre-processing of phenotype and expression data

Phenotypes and expression levels were adjusted for systematic effects by analysis of variance performed with the procedure “Mixed” of the SAS software package (SAS version 9.1 SAS Institute, Cary, NC) before analysing their correlation and by using co-expression network. Sex and RYR genotype was used as a fixed effect, “sire” and “slaughter day” as random effects, and “carcass weight” as a covariate. Subsequently, the residuals of log2-transformed expression intensities (miRNA and mRNA) and muscle phenotype were used for further analysis.

Pearson correlation of miRNA expression level and gene expression level was calculated using 190 individuals; correction for multiple testing was done by controlling the FDR level (q-value according to Storey and Tibshirani, 2003, [94]) at 10%.

### Weighted gene Co-expression network analysis (WGCNA)

A weighted gene co-expression network was constructed for 207 muscle biopsies using the blockwise Modules function from the WGCNA package in R [23]. Residuals of gene expression, after correcting the effect, were used for WGCNA. The blockwise Modules function allows the entire dataset of 11,191 probe sets by mRNA and 675 miRNA to be utilised in the construction of the weighted gene co-expression network.

Extremely outlying individuals were removed from the following analysis based on hierarchically clustered using the average linkage function, and common Euclidean distance. Pearson correlation matrix of all gene-gene comparisons were calculated across all microarrays. Adjacency matrix was then calculated using the correlation matrix of the expression sets. Finally, the Topological Overlap Matrix (TOM) was converted from the adjacency matrix and used to derive a TOM-based distance matrix for the hierarchical clustering of expressions. In the next step, modules of expression profiles, (i.e. sets of genes with high topological overlap) were formed based on hierarchical clustering, with empirically specified minimal module size (30 for gene expression, 10 for miRNA).

According to the WGCNA methodology, rather than traditional distance or correlation based similarity measures, it utilizes the topological overlap matrix *Ω* = [*ω*_*ij*_] (TOM),

$$\omega_{\mathrm{ij}}=\frac{a_{\mathrm{ij}}+\sum_{u}a_{\mathrm{iu}}a_{\mathrm{uj}}}{\min\left\{\sum_{u}a_{\mathrm{iu}},\sum_{u}a_{\mathrm{ju}}\right\}+1-a_{\mathrm{ij}}}{\;,\;a_{\mathrm{ij}}=\left|\mathrm{cor}\left(x_{i},x_{j}\right)\right|}^{\beta }$$

where *x*_*i*_ and *x*_*j*_ are the gene expression profiles of the *i*-*th* and *j*-*th* gene and α_*ij*_ is the adjacency. TOM based distance matrix is a robust and powerful measurement in building co-expression network. Selection of appropriate value for the power β were derived according to the pickSoftThresholding function of the WGCNA package [23,95]. Accordingly, by manually inspecting the fit of the scale free topology model with the candidate β-values for each set of expression profile, minimal β-values giving a coefficient of determination R^2^ higher than 90% were adopted.

Modules were further merged based on the dissimilarity between their “eigengenes”, which were defined as the first principal component of each module. Genes that were not assigned to another module were assigned to the grey module. A threshold of 0.2 for the dissimilarity as recommended by the WGCNA author was used. Module–trait associations were estimated using the correlation between the module eigengene and the phenotype, which allows easy identification of expression set (module) highly correlated to the phenotype. For each expression profile, Gene Significance (GS) was calculated as the absolute value of the correlation between expression profile and each trait; module membership (MM) was defined as the correlation of expression profile and each module eigengene, enabling further identification of key players in the regulation network.

### Gene ontology and pathway enrichment analysis

We performed a gene ontology (GO) enrichment analysis for network modules using the Database for Annotation, Visualization and Integrated Discovery (DAVID, http://david.abcc.ncifcrf.gov/[96,97]. Each analysis was performed using the functional annotation clustering option. Functional annotation clustering combines single categories with a significant overlap in gene content and assigns an enrichment score (ES, defined as the -log10 of the geometric mean of unadjusted *p*-*value*s for each single term in the cluster) to each cluster, making interpretation of the results more straightforward. To assess the significance of functional clusters, we created 22 sets of 11,191 probe sets corresponding to 8,036 genes (size of the average module identified in this study).

### Predicting porcine targets of miRNAs by RNA hybrid and TargetScan

We used two methods to predict the targets of porcine miRNA. First, we predicted targets using the computational software RNAhybrid (http://bibiserv.techfak.uni-bielefeld.de/rnahybrid), which detects the most energetically favourable hybridisation sites of a small RNA within a large RNA [98]. Here, we tested the miRNA probe sets with the following parameters: number of hits per target = 1, energy cutoff = -25 kcal/mol, and maximal internal or bulge loop size per side = 4. Most targets found were located on the 3′-UTR of genes. Second, TargetScan (http://www.targetscan.org) was used to detect target gene candidates based on seed complementarity on UTR database 6.0 and our porcine RefSeq transcript with our miRNA seed sequence [99]. TargetScan was applied considering both conserved and non-conserved targets. The porcine RefSeq transcripts, which derived from 11,191 probe sets that showed consistent expression in porcine muscle, were used as input targets for RNAhybrid and TargetScan.

## Competing interests

The authors declare that they have no competing interests.

## Authors’ contributions

SP and KW conceived the study. SP analysed the microarray data and drafted the manuscript; YD made the WGCNA; FH helped in target prediction analysis. PS, EM, and MS helped in sampling and data collection and drafting the manuscript; KW discussed and contributed to data interpretation and helped in drafting the manuscript. All authors read and approved the final manuscript.

## Supplementary Material

Additional file 1: Figure S1 Dendrogram representing the correlation coefficients between meat quality and carcass traits. Click here for file
