# Supplementary figures and images for: Correlated mRNAs and miRNAs from co-expression and regulatory networks affect porcine muscle and finally meat properties
Source: BMC Genomics. 2013 Aug 5;14:533. doi: 10.1186/1471-2164-14-533 (PMC3750351; doi:10.1186/1471-2164-14-533)

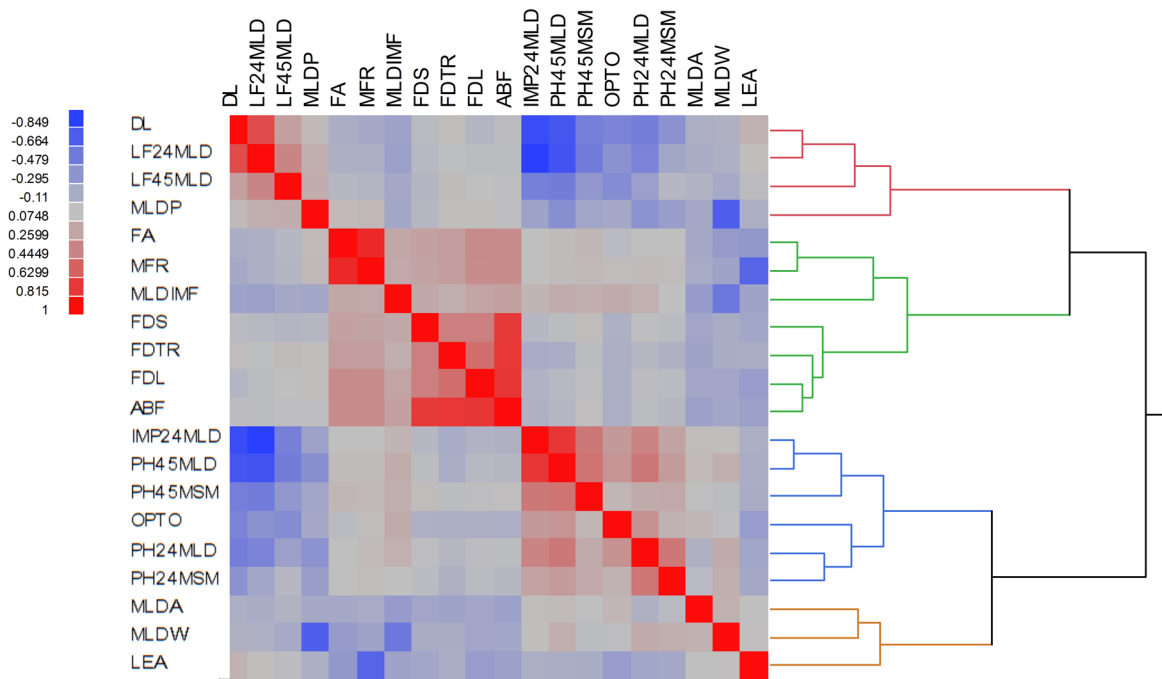

Supplement: Additional file 1: Figure S1 — Dendrogram representing the correlation coefficients between meat quality and carcass traits. [file 1471-2164-14-533-S1.pdf]
